# Fertilization Improves the Yield of *Sapindus saponaria* by Affecting Leaf–Soil–Microbial C–N–P Content and Stoichiometry

**DOI:** 10.3390/plants14091360

**Published:** 2025-04-30

**Authors:** Juntao Liu, Hongbing Yang, Ling Zhou, Shangpeng Zhang, Jie Chen, Xu Wang, Shixiong Wu, Yingyun Gong, Guoqing Zhang, Weihua Zhang, Liming Jia

**Affiliations:** 1 Key Laboratory of Silviculture and Conservation of the Ministry of Education, College of Forestry, Beijing Forestry University, Beijing 100083, China; ljt1120@bjfu.edu.cn (J.L.); zhouling89757@bjfu.edu.cn (L.Z.); wushixiong_1992@163.com (S.W.); oxalis623@163.com (Y.G.); zgq19890615@163.com (G.Z.); 2 Guangdong Provincial Key Laboratory of Silviculture, Protection and Utilization/Guangdong Academy of Forestry, Guangzhou 510520, China; 3 Key Laboratory of Forest Ecology and Environment of National Forestry and Grassland Administration, Ecology and Nature Conservation Institute, Chinese Academy of Forestry, Beijing 100091, China; philyhb@163.com; 4 Beijing Normal University, Beijing 100091, China; zhangsp@mail.bnu.edu.cn; 5 Research Institute of Tropical Forestry, Chinese Academy of Forestry, Guangzhou 510520, China; chenjiecaf@hotmail.com (J.C.); cafwangxu111@126.com (X.W.)

**Keywords:** *Sapindus saponaria*, fertilization, microbial nutrient limitation, C–N–P stoichiometry, TOPSIS

## Abstract

The purpose of this study was to evaluate the effects of different nitrogen (N), phosphorus (P), and potassium (K) fertilization ratios on the carbon (C), N, and P contents and their ecological stoichiometric characteristics in the leaf–soil–microbial system of *Sapindus saponaria* and elucidate their relationship with yield. A “3414” experimental design was employed in a 6-year-old *Sapindus saponaria* woodland located in Fujian Province of China. Fourteen N–P–K fertilization treatments with three replicates were established. Leaf, soil, and microbial samples were collected and analyzed for C, N, and P contents. Redundancy Analysis (RDA), Partial Least Squares Path Modeling (PLS–PM), and the entropy-weighted technique of ranking preferences by similarity to optimal solutions (TOPSIS) were utilized to assess the relationships among variables and determine optimal fertilization strategies. It was found through research that different fertilization treatment methods have a significant impact on both the soil nutrient content and the C, N, and P contents of soil microorganisms. Compared with the control group, soil organic C, total N, and total P, and microbial C, N, and P contents increased by 14.25% to 52.61%, 3.90% to 39.84%, 9.52% to 150%, 6.65% to 47.45%, 11.84% to 46.50%, and 14.91% to 201.98%, respectively. Results from Redundancy Analysis (RDA) indicated that soil organic C, total N, and total P exerted a significant influence on the leaf nutrients. PLS-PM demonstrated that fertilization indirectly affected leaf nutrient accumulation and yield by altering soil properties, with soil total phosphorus and leaf phosphorus being key determinants of yield. Additionally, soil microbial entropy impacted yield by regulating microbial biomass stoichiometric ratios. The entropy-weighted TOPSIS model identified the N_2_P_2_K_2_ treatment (600 kg/ha N, 500 kg/ha P, and 400 kg/ha K) as the most effective fertilization strategy. Optimizing N–P–K fertilization ratios significantly enhances leaf nutrient content and soil microbial biomass C, N, and P, thereby increasing *Sapindus saponaria* yield. This research clarifies the underlying mechanisms through which fertilization exerts an impact on the C–N–P stoichiometry within the leaf–soil–microbial system. Moreover, it furnishes a scientific foundation for the optimization of fertilization management strategies in *Sapindus saponaria* plantations.

## 1. Introduction

As global climate change intensifies and the issue of ecosystem degradation becomes increasingly severe, the efficient management of nutrient cycling within ecosystems has emerged as a crucial area of research. Specifically, the stoichiometric equilibrium of the three key elements—carbon (C), nitrogen (N), and phosphorus (P)—has become a central focus [1,2,3]. In forest ecosystems worldwide, the ecological stoichiometry associated with plants, soils, and microorganisms provides essential insights into ecosystem processes and functions [4,5]. According to the dynamic balance mechanism and growth rate theory, the stoichiometric characteristics of C, N, and P are extensively utilized in assessing plant nutrient balance, allocation of limiting elements, and decomposition processes of litter [6,7,8]. Grasping the correlations and equilibrium conditions of C, N, and P elements in soil and microorganisms is of vital importance. This understanding is key to clarifying the mechanisms of nutrient cycling, particularly in terms of how varying fertilization ratios of N, P, and potassium (K) influence the dynamics of soil nutrients [9,10]. Soil microbial biomass changes are sensitive indicators of soil nutrient alterations and serve as effective indicators for assessing the intensity of soil organic matter metabolism [11]. Previous studies have demonstrated that the utilization of N, P, and K fertilizers not only enhances leaf nutrient content and the survival environment of soil microorganisms but also significantly increases soil microbial biomass and overall soil nutrient levels, thereby affecting the composition structure and variety of microbial communities [12,13,14]. However, the specific roles of different fertilization practices in influencing the ecological stoichiometric properties of C, N, and P in leaf tissues and soil microbiomass remain underexplored.

It has been shown that the C, N, and P stoichiometric traits and plant yields in the plant–soil–microbe system are chiefly impacted by natural factors such as climatic factors, topographic features, altitude, soil properties, and plant traits, and human factors such as fertilizer application, mulching, pruning, and so on [15,16,17,18], with the application of fertilizer having a notably significant influence on the stoichiometric ratios of N, P, and K present in the leaves, soil, and microbial biomass of the plants [15]. Attention should be drawn to the fact that over fertilization reduces the efficiency of fertilizer utilization and affects plant leaves, soil, and microbial biomass C (MBC), N (MBN), P (MBP), and stoichiometric proportions, whereas lesser use of fertilizers or irrational ratios will result in an imbalance of nutrients in plant leaves, soil, and microbials, leading to the reduction in soil fertility, and this subsequently influences plant growth [15,19,20,21,22]. Scientific studies have confirmed that balanced fertilization helps to promote plant leaf, soil, and microbial nutrient balance and improve soil quality, which in turn increases crop yields and improves fruit quality [23]. While high P or K input significantly impacted the diversity in combination with the composition of soil bacterial communities while decreasing differences in soil C, N, and P and their stoichiometric ratios, high N input significantly increased MBC and MBC:MBN when compared to low N input over time under various fertilization conditions [24]. A number of studies have discovered that the addition of P significantly decreases the N:P and C:N ratios of plant, soil, and microbial biomass, with little effect on the C:N ratio [7]. In addition, N nitrogen addition significantly reduced plant and soil C:N but increased plant N:P ratios and soil microbial carbon and phosphorus limiting [3]. In addition, soil nutrient status has important direct effects on plant leaf C, N, and P content and stoichiometry [25]. Meanwhile, plant leaf C and N content and C:N were significantly correlated with soil C and N content [26]. Reasonable fertilization management can maximize crop yield [23]. In actual production, due to the lack of scientific rationing, ratios of N, P, and K are often unbalanced, which affects plant nutrient uptake and utilization, reduces yield and quality, and increases the risk of nutrient loss and environmental pollution. Therefore, reasonable N, P, and K ratios can significantly promote plant fruit yield, and there is an urgent need to explore scientific fertilization rationing strategies, which are essential for enhancing sustainable plant development.

In recent years, the methods currently commonly used for fertilizer evaluation include the affiliation function value method and the principal component analysis method [27]. The TOPSIS method is a multi-attribute decision-making method of ideal objective similarity and approximation of ideal solution ranking method, while the entropy-weighted TOPSIS model is an improved model combining the entropy-weighted method and the TOPSIS model, which is the most recent research result in the comprehensive evaluation method [27,28,29]. It has been applied to the fertilization evaluation of different plants such as *Panax notoginseng* [28] and *Mangifera indica* [30], but we have not yet seen any report on the evaluation of the C, N, and P content of leaf–soil–microbial C, N, and P content and stoichiometric ratio of *Sapindus saprophyllus*, as well as the yield, by using the entropy-weighted TOPSIS method. Therefore, an entropy-weighted TOPSIS comprehensive evaluation system was constructed to systematically quantify the C, N, and P coupling characteristics and yield response mechanism in the leaf–soil–microbe system of *Sapindus mukorossi* and to break through the limitations of the traditional single-factor evaluation through the optimization of multi-dimensional index weights and relative proximity ranking to provide scientific bases for *Sapindus mukorossi*’s ecocultivation and nutrient precision management. It provides a scientific basis for the ecological cultivation and precise management of nutrients in *Sapindus indica*.

*Sapindus saponaria* is an energy plant extensively spread across the tropical and subtropical areas of China, with multiple functions such as biomass energy production, ecological restoration, and landscaping [27,31,32,33]. In Fujian Province, one of its primary cultivation areas, the planting area has reached 20,000 hectares. Traditional fertilization practices, characterized by irrational fertilizer structures and unscientific fertilizer mixing, often lead to severe environmental problems such as soil nutrient imbalance, acidification, and groundwater pollution, which seriously affect *Sapindus saponaria* yield and sustainable development [27]. Although previous studies have shown the effects of N, P, and K fertilization on *Sapindus saponaria* growth, photosynthetic physiology, and soil nutrient balance, systematic research is lacking about the combined effects of different fertilization treatments on yield concerning leaf, soil, and microbial mass C, N, and P, and stoichiometric traits. In this context, the present study employs a “3414” experimental design to explore the effects of varying N–P–K fertilization ratios on the C, N, and P contents and their stoichiometric characteristics in the leaf–soil–microbial system of a 6-year-old *Sapindus saponaria* plantation. Utilizing Redundancy Analysis (RDA), Partial Least Squares Path Modeling (PLS–PM), and entropy-weighted TOPSIS, this study aims to (1) quantify the effects of various N–P–K ratios on the C, N, and P contents of *Sapindus saponaria* leaves, soil, and microorganisms; (2) reveal the relationship regarding the relationship among soil and microbial biomass N, P, and K content and stoichiometric characteristics with leaf nutrient profiles; (3) verify whether fertilization indirectly affects microbial biomass by altering soil nutrients, thereby improving *Sapindus saponaria* yield; and (4) develop a scientific nutrient management plan for *Sapindus saponaria* plantations to promote sustainable development and productivity. These objectives aim to provide comprehensive insights into optimizing fertilization strategies for *Sapindus saponaria,* enhancing yield, and maintaining ecosystem health.

## 2. Results

### 2.1. Effects of Different Fertilization Treatments on Carbon, Nitrogen, Phosphorus, and Stoichiometry of Sapindus saponaria Leaves

**Figure 1:**
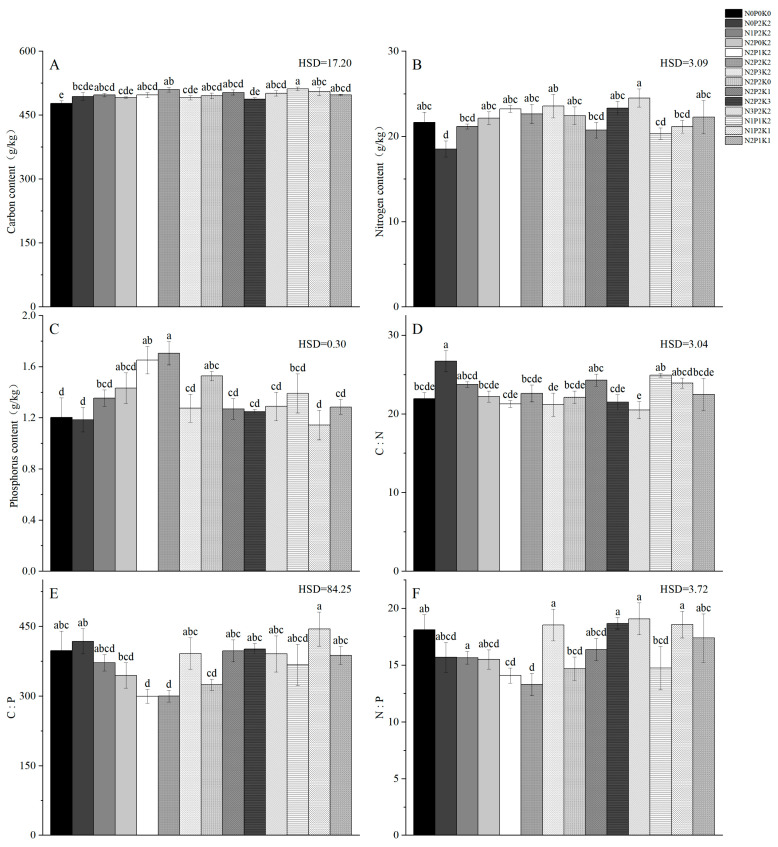
Characteristics of organic C, total N, and total P contents and their stoichiometric ratios of *Sapindus saponaria* soils under different N–P–K fertilization treatments: (**A**) leaf C content, (**B**) leaf N content, (**C**) leaf P content, (**D**) LC:LN, (**E**) LC:LP, and (**F**) LN:LP. Different lowercase letters indicate significant differences between fertilization treatments at the 0.05 statistical significance level.

Compared with the CK treatment group, different fertilization treatments significantly increased leaf C, N, and P contents (Figure 1), which took values ranging from 2.74 to 7.21%, −14.44 to 13.16%, and −5 to 41.67%, respectively, and leaf C and P contents under each fertilization treatment showed a tendency of increasing and then decreasing with the additions of applied N, P, and K at the levels of P_2_K_2_, N_2_K_2_, or N_2_P_2_; all of them in the leaf C and P content reached the highest in the N_2_P_2_K_2_ treatment. Compared with N_0_P_2_K_2_, N_2_P_0_K_2_, and N_2_P_2_K_0_ treatments, the leaf organic C and total P contents of treatment N_2_P_2_K_2_ were increased by 3.19% and 44.92%, 3.79% and 19.58%, and 2.87% and 11.76%, respectively, and there was a significant difference between treatments N_2_P_2_K_2_ and N_0_P_2_K_2_ and N_2_P_0_K_2_ and N_2_P_2_K_0_. Moreover, one-way ANOVA showed that different fertilization treatments had a significant effect on leaf C:P and N:P stoichiometry, while there was no significant effect on its leaf C:N.

### 2.2. Soil Carbon, Nitrogen, and Phosphorus Contents, and Stoichiometric Ratios Under Different Fertilization Treatments

**Figure 2:**
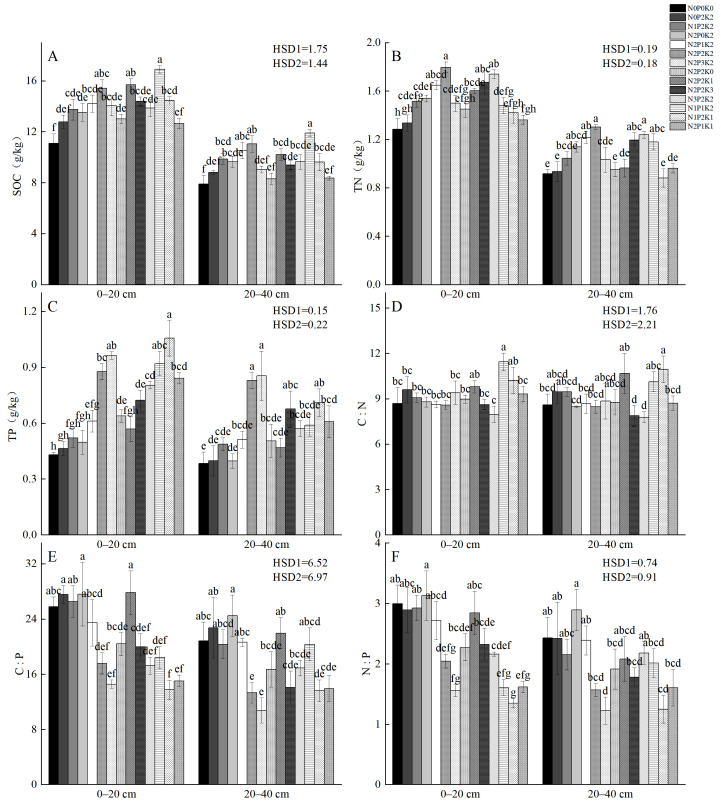
Characteristics of SOC, TN, and TP contents and their stoichiometric ratios of *Sapindus saponaria* soils under different N–P–K fertilization treatments. (**A**) SOC, organic carbon; (**B**) TN, total nitrogen; (**C**) TP, total phosphorus; (**D**) C:N, carbon/nitrogen ratio; (**E**) C:P, carbon/phosphorus ratio; (**F**) N:P, nitrogen/phosphorus ratio. HSD1: 0–20 cm. HSD2: 20–40 cm. A significant difference at the 0.05 level exists between fertilization treatments denoted by different lowercase letters.

Compared to CK, soil organic carbon (SOC), total nitrogen (TN), and total phosphorus (TP) contents increased by 14.25% to 52.61%, 3.90% to 39.84%, and 9.52% to 150% given different fertilization strategies (Figure 2). In 0–20 cm and 20–40 cm soil layers, SOC and TN contents presented a trend of growth followed by decline with the increase in N, P, and K application. The increases in SOC, TN, and TP were more obvious in the 0–20 cm layer than in the 20–40 cm layer, with P content showing the greatest increase across soil layers.

Soil C:N, C:P, and N:P ratios under different fertilization treatments ranged from 7.97 to 11.44, 13.76 to 27.81, and 1.35 to 3.13, respectively (Figure 2). Compared to CK, the soil C:N ratio tended to increase in all treatments except N_2_P_1_K_2_, N_2_P_2_K_2_, N_2_P_2_K_3_, and N_3_P_2_K_2_. Soil N:P ratios showed significant differences among treatments, while N:P ratios showed no significant differences.

### 2.3. Microbial Carbon, Nitrogen, and Phosphorus Contents, and Stoichiometric Ratios Under Different Fertilization Treatments

**Figure 3:**
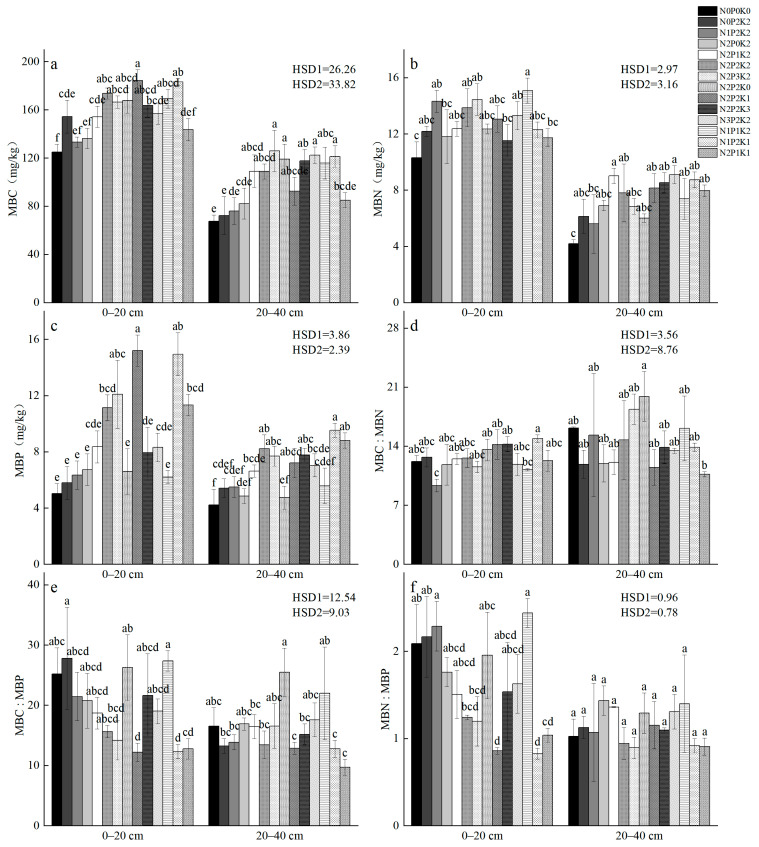
Characteristics of MBC, MBN, and MBP and stoichiometric ratio of *Sapindus saponaria* soil under different N–P–K fertilization treatments. (**a**) MBC: microbial carbon content; (**b**) MBN: microbial nitrogen content; (**c**) MBP: microbial phosphorus content; (**d**) MBC:MBN: microbial carbon to nitrogen ratio; (**e**) MBC:MBP, microbial carbon to phosphorus ratio; (**f**) MBN:MBP, microbial nitrogen to phosphorus ratio. At the 0.05 significance level, distinct lowercase letters denote substantial differences among fertilization treatments.

Compared to the control treatment (CK), soil MBC, MBN, and MBP contents increased by 6.65–47.45%, 11.84–46.50%, and 14.91–201.98%, respectively, under fertilization treatments. At P_2_K_2_ and N_2_P_2_ levels, soil MBN and MBP contents initially increased and then decreased with increasing N and K application, while MBP content steadily increased with increasing P application in the N_2_K_2_ treatment. Soil microbial biomass ratios (MBC:MBN, MBC:MBP, and MBN:MBP) ranged from 9.33 to 14.89, 12.19 to 27.81, and 0.82 to 2.44, respectively (Figure 3). One-way ANOVA indicated significant differences in soil MBC, MBN, and MBP contents among treatments (*p* < 0.05), but no significant differences in their stoichiometric ratios.

### 2.4. Leaf–Soil–Microbial Biomass C, N, P Contents, and Stoichiometric Characteristics

**Figure 4:**
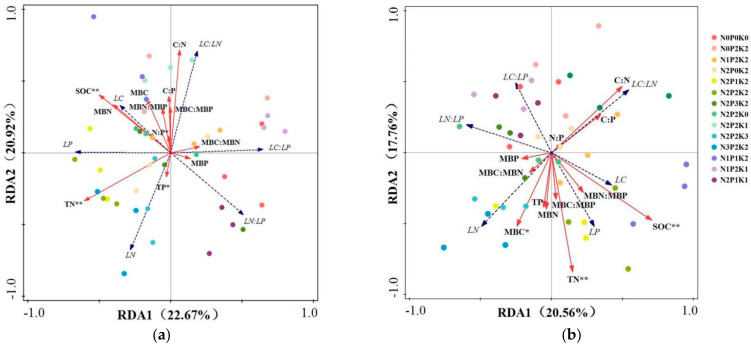
RDA of soil and microbial biomass C, N, P, and stoichiometric ratios of *Sapindus saponaria* under different fertilization treatments: (**a**) 0 to 20 cm soil layer and (**b**) 20 to 40 cm soil layer.

In the 0–20 cm soil layer, the first axis and second RDA axis explained 22.67% and 20.92% of the variation in leaf nutrient characteristics, respectively, collectively accounting for 43.59% of the variation (Figure 4a). Leaf N content showed a positive relationship to leaf P content and leaf N:P ratio and soil N and P contents and had a negative correlation with soil N:P ratio and leaf C:N ratio. Leaf C:P ratio was positively correlated with MBP content and MBC:MBN, whereas leaf N:P ratio was negatively associated with MBC:MBP.

Within the soil layer from 20 to 40 cm, the two RDA axes explained 38.32% of the variation in leaf nutrient characteristics (Figure 4b). Soil TP, TN, and SOC contents significantly affected leaf nutrient characteristics (*p* < 0.05), with SOC having the greatest impact (Table S1).

**Figure 5:**
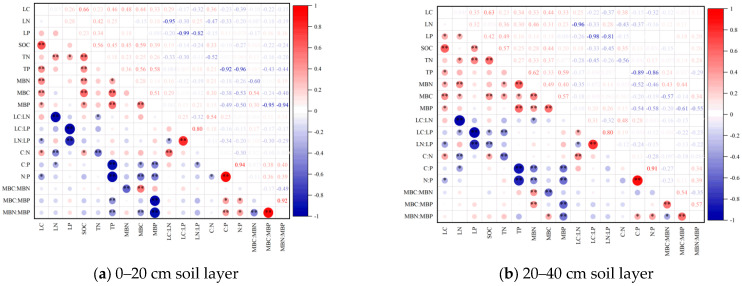
Correlations between carbon, nitrogen, and phosphorus contents of leaves, soil, and microbiomass of *Sapindus saponaria* in 0–20 cm (**a**) and 20–40 cm (**b**) soil layers. LC: leaf blade carbon content; LN: leaf blade nitrogen content; LP: leaf blade phosphorus content; SOC: organic carbon; TN: total nitrogen; TP: total phosphorus; C:N: SOC to TN ratio; C:P: SOC to TP ratio; N:P: TN to TP ratio; LC:LN: leaf blade carbon to nitrogen ratio; LC:LP: leaf blade carbon to phosphorus ratio; LN:LP: leaf blade nitrogen to phosphorus ratio. MBC: microbial biomass carbon content; MBN: microbial biomass nitrogen content; MBP: microbial biomass phosphorus content; MBC:MBN: microbial biomass carbon to nitrogen ratio; MBC:MBP: microbial biomass carbon to phosphorus ratio; MBN:MBP: microbial biomass nitrogen to phosphorus ratio.

As can be seen from Figure 5, in 0–20 cm and 20–40 cm soil layers, leaf C content showed highly significant positive correlations with SOC, TP, and soil MBC, MBN, MBP, and soil C:N, and significant negative correlations with soil N:P and leaf LN:LP. The leaf LC:LN exhibited a significant positive correlation with the soil C:N. Meanwhile, it showed a significant negative correlation with the leaf N content. Soil N:P was significantly positively correlated with MBN:MBP and soil C:P and significantly negatively correlated with soil TP, MBC content, and soil C:N. Highly significant positive correlations were detected among the soil MBC, MBN, and MBP contents.

### 2.5. Multi-Objective Decision-Making and Evaluation Processes Leveraging the Entropy-Weighted TOPSIS Approach

The entropy-weighted TOPSIS model was used to comprehensively evaluate each fertilization treatment based on multiple indicators (Table 1). In the 0–20 cm soil layer, the order of advantages and disadvantages of the top 5 treatments of *Sapindus saponaria* obtained by TOPSIS was N_2_P_2_K_2_ > N_1_P_2_K_1_ > N_2_P_3_K_2_ > N_2_P_2_K_0_ > N_2_P_1_K_2_, of which the best comprehensive evaluation of the soil layer of 20–40 cm and 0–20 cm was the N_2_P_2_K_2_ treatment. Therefore, the N, P, and K fertilization of 600 kg/ha, 500 kg/ha, and 400 kg/ha in the field can effectively improve the comprehensive indexes of *Sapindus saponaria*, respectively.

### 2.6. Analysis Using Partial Least Squares Path Model (PLS–PM)

**Figure 6:**
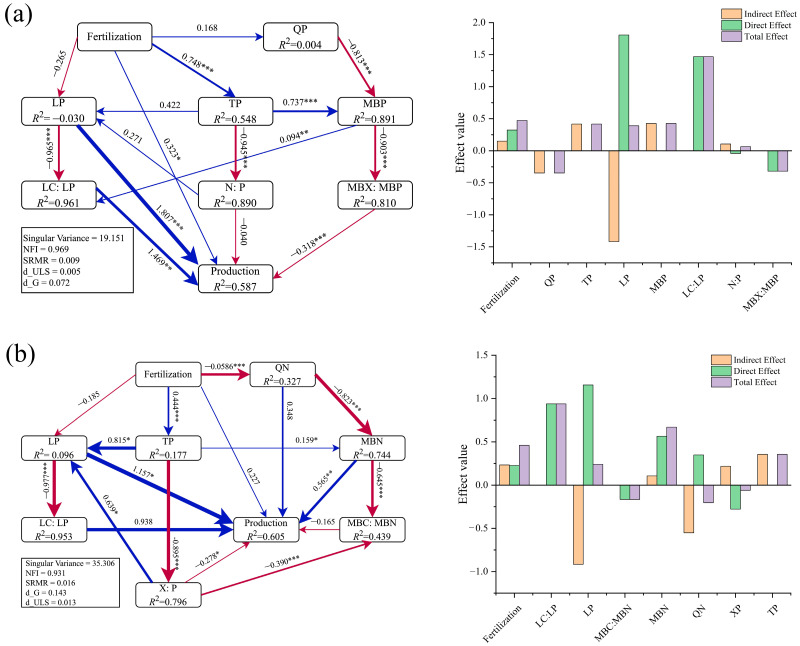
PLS–PM analysis for 0 to 20 cm and 20 to 40 cm soil layers: (**a**) 0–20 cm layer soil and (**b**) 20–40 cm layer soil. Fertilization denotes the amount of fertilizer applied. QN and QP represent microbial entropy nitrogen and phosphorus; MBX:MBP, MBC:MBP, and MBN:MBP ratios; and X:P: C:P, and N:P ratios. Production denotes the yield of the fruit. Blue lines indicate positive impacts, and red lines indicate negative impacts. Straight line thickness indicates relative magnitude of impact. The table of numbers on the lines were path coefficients. *, *p* < 0.05; **, *p* < 0.01; ***, *p* < 0.001. *R*^2^ denotes adjusted fit.

From the PLS–PM about the yield of *Sapindus saponaria*, it was observed that the model fitness of soil layers at depths of 0–20 cm (topsoil) and 20–40 cm (subsoil) met the acceptable requirements and explained 58.7% and 60.5% of the variation in the yield of *Sapindus saponaria*, respectively (Figure 6), with fertilizer, LP, LC:P, and MBX:MBP directly and significantly affecting its yield. It was easy to find that leaf C:P affected the highest total effect on *Sapindus saponaria* yield and leaf P content had the greatest direct or indirect effect on *Sapindus saponaria* yield. In the topsoil case, there were two distinct pathways affecting the yield of its saprophytes (Figure 6a; Table S2) as follows: ① fertilizer application directly positively affects the yield, and ② fertilizer application ultimately negatively affects the yield by directly positively affecting the TP content, which in turn positively affects the MBP content, which in turn negatively affects the MBC:MBP and MBN:MBP. In addition to this, fertilization also affects the leaf P content and its C:P by influencing the TP content, which in turn positively affects the yield. In the subsoil case (Figure 6b; Table S2), there were four distinct pathways affecting the yield of its saprophytes as follows: (1) fertilizer application positively affects its yield by positively affecting TP, which in turn positively affects LP; (2) fertilizer application negatively affects its yield by positively affecting TP, which in turn negatively affects C:P and N:P; (3) fertilizer application negatively affected QN by affecting, in turn, MBN content, which in turn positively affected yield; and (4) fertilizer application affected MBN content by positively affecting TP content, which in turn positively affected yield.

In addition, the yield of *Sapindus saponaria* is regulated by several factors, and long-term input of inorganic fertilizer directly reduces its yield by decreasing leaf P content on the one hand, and suitable fertilization facilitates the increase in soil TP content in order to increase leaf P content, which significantly improves the yield of *Sapindus saponaria*. On the other hand, by affecting microbial entropy to influence the microbial biomass content and its stoichiometric ratio, which directly or indirectly acted on the yield.

## 3. Discussion

### 3.1. Characterization of C, N, and P Contents and Their Stoichiometric Ratios in Leaves

Numerous studies indicate that N and P stand out as the two primary limiting factors constraining plant growth. Concurrently, the leaf N:P ratio is extensively adopted as a crucial indicator for gauging plant nutrient limitations. The magnitude of plant leaves’ N:P ratio reflects the relative limitation of N or P at the community level. Changes in this ratio have the potential to initiate modifications in plant functional traits, vegetation composition, and plant diversity [34,35,36]. There is a general consensus that plant leaves with N:P ratios lower than 14 indicate that the plant may be N-limited; N:P ratios higher than 16 indicate P-limitation; and if the N:P ratios of plant leaves are between 14 and 16, the plant may be limited by both N and P elements [37]. Also, high C:N ratios reflect that N may be a limiting factor, while low C:N ratios indicate relative N abundance, which may affect plant C balance [38,39]. In the context of this research, the mean value of the N:P ratio of *Sapindus saponaria* leaves was significantly higher than the mean value of 16.30 for Chinese plants and the global mean range of 12.7–13.8 [40,41], and the overall P-limiting characteristics were demonstrated, which indicated that the P-limiting intensity of the *Sapindus saponaria* leaves was greater in this forest stand.

Even though utilizing P fertilizer can boost plant leaf P uptake and enhance the resistance to P stress, it cannot offset the negative effects of inefficient P fertilizer utilization, as well as the limited ability to effectively mitigate the intensity of P limitation, which is closely bound up with the specific types of fertilizer application, plant characteristics, regional differences, etc. [42,43,44]. Specifically, leaf N and P contents in the study area were 18.52–23.55 and 1.14–1.71, respectively (Figure 1). These findings are consistent with the analysis of 753 species by Han et al. [37] and further confirm the higher degree of P-limitation exhibited by plant leaves in the subtropics. This effect of high leaf N:P ratio can be attributed to the low natural P content and strong N deposition in the soils of subtropical regions of China [37]. At the ecosystem scale, there is a coupling relationship between plant leaf P content and soil P content [45,46], which agrees with the outcomes presented herein. In response to leaf P limitation, soil P content can be effectively increased by fertilization, which in turn increases plant leaf P content, thereby reducing the leaf N:P ratios and mitigating the intensity of P limitation [47]. In conclusion, moderate fertilization can significantly increase leaf C, N, and P contents to alleviate plant leaf P-limitation and better maintain plant growth function.

### 3.2. Characterization of Soil–Microbial C, N, and P Contents and Stoichiometric Ratios

The findings of this research demonstrated that fertilization significantly influenced the nutrient levels and fertility augmentation of *Sapindus saponaria* growing soils. Specifically, fertilization significantly increased soil C, N, and P contents and effectively improved soil fertility. The result here is in accordance with those of other tree species [23,48]. The reasons for the efficiency of fertilizer application (increased yield, improved fertility, etc.) may lie in the fact that fertilizer application directly increases soil C, N, and P contents, and applying fertilizer stimulates plant growth and boosts the amount of plant residues incorporated into the soil, thus indirectly increasing the accumulation of soil nutrients [49]. Compared with the no fertilization treatment, TP content displayed an upward trend with the increase in P fertilizer at different levels of P fertilizer addition. This is due to the fact that P easily combines with Al^3+^ and Fe elements triggered by soil acidification and then transforms into low-soluble compounds, which can effectively increase soil effective P content [50]. Furthermore, findings indicated that SOC content increased at first and later decreased when N, P, and K were applied to the soil. According to the “stoichiometric decomposition theory” formulated by Hessen et al. [51], the imbalance of stoichiometric ratios in the soil was alleviated by N inputs, and microbial activity could be increased, thus accelerating SOC decomposition. Decomposition rates and microbial activity were highest when C and N inputs corresponded to microbial stoichiometric C:N ratios. When fertilizer was applied in excess, it is likely that it exerted an influence on the richness and activity of the soil microbial community. This, in turn, had an impact on the biodegradation process of SOC sources, ultimately leading to a decreased SOC content [52]. This indicates the importance of moderate fertilization in maintaining the balance of SOC content.

C and nutrient stoichiometric ratios (C:N, C:P, and N:P) in soil act as vital indices of the efficiency of soil nutrients [53,54]. The results of this study yielded that the C:N, C:P, and N:P of saprophytic soils under different fertilization treatments ranged from 7.97 to 11.44, 13.76 to 27.81, and 1.35 to 3.13, respectively, and the average C:N ratio of the soils in the study area, which was 9.21, was notably lower than the corresponding ratio of soils across China, which stood at 11.9. This reflects the higher rate of decomposition of soil organic matter as well as the higher potential for N accumulation in the investigated region. The average C:P value of the soil was 21.13, which was considerably below the average level (136) across China [55]. This suggests that microbial mineralization of soil organic matter has a high potential for releasing P, which is conducive to promoting P uptake by plants [56]. In addition, the average soil N:P ratio was measured at 2.31, which was substantially lower than both the global average ratio of 5.9 and the average ratio of 5.2 observed in China [55], which also suggests that soils in this region are more significantly P-limited.

In addition, fertilization significantly affected soil microbial biomass and its stoichiometric ratio characteristics, and soil TP and SOC contents were the primary determinants contributing to the variations in soil microbial biomass. The mean ratios of MBC:MBN, MBC:MBP, and MBN:MBP in *Sapindus saponaria* soils under various fertilization treatments were 12.49, 19.63, and 1.61, respectively. Specifically, the MBC:MBN ratio significantly exceeded the global average (typically, 8.3–10) [57], while the MBN:MBP ratio fell below the global average level (typically, 3.5–8.0) [5]. These imbalances suggest a fungal-dominated microbial community structure in the study area, as fungi generally exhibit higher C:N and lower N:P ratios compared to bacteria [5,57]. Since a higher MBC:MBN ratio usually reflects a fungal-dominated community structure, fungi tend to grow and metabolize in high C:N ratio environments more than bacteria, which also suggests that microorganisms are somewhat P-limited in terms of nutrient limitation [5,57,58]. Overall, under various fertilization treatments, the P content of saprophytic soils experienced a more significant increase compared to other indicators, thus indicating that the nutrient limiting factor for soil microorganisms is P content [5,58]. Given that the analysis within this study did not include the soil enzyme activities and the traits of microbial community structure, the intrinsic regulatory mechanisms between the soil–microbial biomass and its stoichiometric ratio characteristics and its other microbial properties still need to be explored in depth in the follow-up.

### 3.3. Influence of Fertilization Regimes on Fruit Yield of Sapindus saponaria

The effectiveness of N, P, and K fertilizers in increasing crop yields is in general agreement with the conclusions reached in most studies [23,59]. This is due to the fact that balanced fertilization enhances soil nutrient availability and improves plant growth conditions. The findings of this study indicated that the C, N, and P contents present in both the plants and soil microorganisms were better in the N_2_P_2_K_2_ treatment using the entropy-weighted TOPSIS model (Table 1). It was also found that N_2_P_2_K_2_ treatment was the optimal fertilization rate for fruit yield enhancement in *Sapindus saponaria* (Figure S1), but excessive fertilization led to yield decline instead, probably because excess nutrients may lead to soil compaction and nutrient imbalance when fertilization exceeds the threshold, thus inhibiting the plant root growth and nutrient uptake, which ultimately led to yield decline, and this phenomenon is applicable to other crops as well [60]. Therefore, when fertilizer is applied within reasonable limits, the supply of N, P, and K in the soil increases, which in turn promotes higher fruit yields. Additionally, moderate fertilization helps to enhance plant resistance, reduce pests and diseases, and further increase yield.

Long-term balanced fertilization (such as N_2_P_2_K_2_) significantly improves the structure of the microbial community by increasing the contents of SOC, N, and P. It also enhances MBC and metabolic activity while reducing metabolic entropy (CO_2_ emission per unit of biomass). This optimization can promote soil nutrient mineralization and the efficiency of plant nutrient uptake, thus supporting high fruit yields [61]. Some studies have emphasized that in calcareous alluvial soils deficient in P, rational application of P is a prerequisite for improving the utilization efficiency of N fertilizers [62]. This is consistent with the contribution of P to yield in the N_2_P_2_K_2_ treatment of this study, and it explains that excessive application of N may lead to a relative deficiency of P, inhibiting the symbiosis of arbuscular mycorrhizal fungi (AMF) and further limiting the nutrient uptake by the roots. In addition, the suitable soil pH value for plants of the *Sapindus* is 5–6. If excessive fertilization leads to a decrease in the soil pH value in the experimental area, thus resulting in severe soil acidification, it may directly inhibit root development and microbial activities, resulting in a decline in yield [63]. The results of multiple studies have shown that the combined application of organic fertilizers can enhance the stability of soil aggregates and the complexity of the microbial network, which may further improve the sustainability of yield [61,62]. Based on the limitations of current research, on the basis of optimizing existing N–P–K inorganic fertilizer ratios, research will be conducted on the coupled application of organic and biological fertilizers. The focus is on analyzing soil–plant system nutrient cycling characteristics, microbial community structure responses, and synergistic yield-quality improvement mechanisms under fertilizer synergism. By constructing a three-in-one “inorganic–organic–biological” fertilization management model, this study aims to provide multi-dimensional solutions for the sustainable development of *Sapindus*.

### 3.4. Plant–Soil–Microbe Coupling

From the results of RDA and PLS-PM, it was concluded that the TP and SOC contents of the soil substantially influenced the yield of *Sapindus saponaria*, MBN or MBP content, and microbial activity. One possible explanation for this occurrence is the rapid escalation of the soil’s TP levels and SOC content after fertilization, which can provide raw materials for soil microbial activities and accelerate the absorption and conversion of soil nutrients. The correlation analysis presented evidence that the MBC, MBN, and MBP of *Sapindus saponaria* soil exhibited a highly significant positive correlation with SOC, TN, and TP contents (*p* < 0.01). This result corresponds with the conclusions drawn from most investigations into woody plants, where fertilization promoted soil nutrient accumulation in the 0–20 cm and 20–40 cm soil layers, affecting *Sapindus saponaria* yield mainly through changes in soil P content and its N:P, leaf P content and its C:P ratio, and microbial entropy N or P (Figure 6. When compared to the 0–20 cm soil layer, the fluctuations in soil nutrient content and its stoichiometric ratios within the 20–40 cm soil layer, along with the variations in leaf P content and the C:N ratio of leaves corresponding to the 20–40 cm soil layer, provided a more substantial explanation for the changes in the yield of *Sapindus saponaria*, but the difference in the degree of explanation between the two layers was not significant. This may be due to the fact that *Sapindus saponaria* has a deeper root system, so that the main nutrients absorbed by it mainly come from the deeper soil; soil nutrient status of the 20–40 cm soil layer may better reflect the long-term nutrient supply status of the plant, thus exerting a more pronounced influence on the leaf nutrient status and yield; 20–40 cm soil layer is more capable of regulating the rate of nutrient release due to the deep regulation of soil stoichiometric equilibrium; soil enzyme activities show differences in different soil layers [64,65], but the activities of P-related metabolizing enzymes in their two soil horizons may not be so different as to affect soil P levels to a similar degree and have a similar effect on yields.

In conclusion, P content in soil and leaves serves as a restricting factor in determining crop yield [66,67,68], and the key reason for the changes in crop yields lies in the soil nutrient status, especially the P content within the soil, while soil microbial biomass affects yield indirectly by influencing soil enzyme activity and nutrient levels and indirectly affects yield [69]. However, exorbitant fertilization practices have the potential to trigger severe soil degradation, thereby throwing the soil’s nutrient structure out of balance and subsequently impeding the growth and development of the plant root system, reducing nutrient and water uptake capacity, and indirectly inhibiting yield. Soil MBN and MBP content are key factors in regulating the main pathway of *Sapindus saponaria* yield, and TP content had a significant direct effect on soil microbiomass (Figure 6), suggesting that soil environmental factors jointly driving the shift of MBC, MBN, and MBP were generated under the distinct fertilization regimens. This aligns generally with the results of investigations conducted by other scholars [70,71,72].

## 4. Materials and Methods

### 4.1. Overview of the Experimental Site

Our experimental site was in Jianning County, Fujian Province (116°47′20″ E, 26°40′3″ N), which belongs to the subtropical monsoon climate zone, with a cold climate in winter and a large temperature difference between day and night in summer. The soil type of the experimental site was sandy clay loam. The values of soil physicochemical properties in the background are presented in an article published by our team [27]. The average plant height and ground diameter within the stand of the asexual line ‘Yuanhua’ were 2.38 m and 6.97 cm. The location of the experimental site and experimental design of different N–P–K treatments are presented in the Supplementary Materials (Figure S2).

### 4.2. Experimental Design

The experimental materials were planted in 6 years old Sapindus “Yuanhua” asexual forest stands. A “3414” randomized block design was used, including 3 factors (N, P, and K), 4 levels for each factor, a total of 14 combinations of treatments, 3 replications, and a total of 42 plots. Here, 0 level was for no fertilizer application and served as a control. Fertilizer application rates are listed in Table 2. The treatment plots were separated by isolation rows. Fertilizer was applied three times a year, on April 10 (during flowering, 30% of the total fertilizer application), July 20 (during fruiting, 30% of the total fertilizer application), and November 1 (after harvest, 40% of the total fertilizer application).

Fertilizer was applied by furrow application, mixing the fertilizer well into the soil and covering it immediately. Maintenance and management were the same for all plots except for the fertilizer treatment. The test fertilizers were urea (46.0% N), calcium superphosphate (12.0% P_2_O_5_), and potassium sulfate (60.0% K_2_O).

### 4.3. Sample Collection and Measurement

Soil sampling and determination: Three sampling points were randomly selected in each plot during the fruit expansion period of *Sapindus saponaria*. Soil samples were collected from 0 to 20 cm and 20 to 40 cm soil layers at each sampling site. After removing impurities and sapodilla root residues from the collected soil samples, they were divided into two parts. One part was put into a self-sealing bag and brought back to the laboratory to dry naturally and pass through a 2 mm sieve for the determination of soil chemical properties; the other part was put into a 50 mL centrifuge tube, and the soil samples were preserved in an icebox and brought back to the laboratory at 4 °C for low-temperature storage for the determination of soil microbial biomass. SOC was determined by the potassium dichromate oxidation–external heating method [73]; soil nitrogen was determined by the Kjeldahl method; and soil total phosphorus was determined by the molybdenum-antimony colorimetric method. MBC and MBN were determined by chloroform fumigation–K_2_SO_4_ extraction, and soil MBPs was determined by chloroform fumigation–NaHCO_3_ leaching [49,74]. Yield: after harvesting at maturity, the plants were weighed on an electronic scale, and the average yield per plant was calculated.

### 4.4. Calculation of Leaf–Soil–Microbe-Related Indicators

The C:N ratio is the ratio of SOC content to TN content, the soil C:P ratio is the ratio of SOC content to TP content, and the soil N:P ratio is the ratio of TN content to TP content. Leaf carbon to nitrogen ratio (LC:LN) is the ratio of organic C content to total N content, carbon to phosphorus ratio (LC:LP) is the ratio of organic C content to total P content, and nitrogen to phosphorus ratio is the ratio of leaf total N content to total P content. Soil microbial entropy carbon (QC) is calculated as SOC content over MBC content, microbial entropy nitrogen (QN) is calculated as TN content over MBN content, and microbial entropy phosphorus (QP) is calculated as TP content over MBP content [75].

### 4.5. Multi-Objective Decision Making and Evaluation Based on Entropy-Weighted TOPSIS Approach

The entropy-weighted TOPSIS method involves both the entropy-weighted method and the TOPSIS method; the entropy-weighted method first standardizes each index and calculates the weight value of each evaluation index, and then further calculates the weight value of entropy weight and uses the weight value of entropy weight to multiply by the standardized original data to obtain the new data value. Using the new data values for the TOPSIS method for calculation, the Ci value of each ideal solution of the proximity procedure of each evaluation object is finally obtained so as to judge and measure the superiority and inferiority ranking of the evaluation object. The specific calculation process can be referred to in the literature [29,30]. The analysis process is as follows:

(1) An evaluation index matrix of leaf and soil C, N, and P contents, as well as yield attributes of *Sapindus* indica under different fertilization treatments, was established as follows.

(1) $$X=\left[\begin{array}{ccccc} X_{11} & \cdots & X_{1j} & \cdots & X_{1m}\\ \cdots & & \cdots & & \cdots \\ X_{i1} & \cdots & X_{ij} & \cdots & X_{im}\\ \cdots & & \cdots & & \cdots \\ X_{n1} & \cdots & X_{nj} & \cdots & X_{nm} \end{array}\right]$$

where X_ij_ is the j indicator value of the i treatment of the original data (i = 1, …, n; j = 1, …, m), n is the number of treatments (n = 14), and m is the number of evaluation indicators (m = 10).

(2) Standardization of evaluation indicators to harmonize the types and dimensions of each indicator, with the following formula.

For positive indicators, use the following formula:

(2) $$X_{ij}^{\prime }=\frac{X_{ij}-max\left(X_{1j},X_{2j},\ldots ,X_{ij}\right)}{\max\left(X_{1j},X_{2j},\ldots ,X_{ij}\right)-min(X_{1j},X_{2j},\ldots ,X_{ij})}+1$$

For negative indicators, use the following formula:
  (3) $$X_{ij}^{\prime }=\frac{\max\left(X_{ij},X_{2j},\ldots ,X_{ij}\right)-X_{ij}}{\max\left(X_{ij},X_{ij},\ldots ,X_{ij}\right)-min(X_{1j},X_{2j},\ldots ,X_{ij})}+1$$
  where X_ij_ is the j indicator value for the i treatment (i = 1, …, n; j = 1, …, m) and X’_ij_ is the normalized X_ij_.

(3) The proportion of the j indicator represented by the i treatment (P_ij_) is calculated as follows:

(4) $$P_{ij}=\frac{X_{ij}^{\prime }}{\sum_{i=1}^{n}X_{ij}}$$

(4) The entropy value e_j_ of the j indicator is calculated as follows:

(5) $$e_{j}=-\frac{\sum_{i=1}^{n}P_{ij}\ln\left(P_{ij}\right)}{\ln n}$$

(5) The coefficient of variation g_j_ for the j indicator is calculated as follows:

(6) $$g_{i}=1-e_{j}$$

(6) The weight W_j_ of the j indicator is calculated as follows:

(7) $$W_{j}=\frac{g_{i}}{\sum_{i=1}^{m}g_{i}}$$

(7) A weighted normalized decision matrix (R) is formed from the normalized decision matrix X = (X’_ij_)14 × 10 and the weight vector W = (w1, w2, w3, …, w14).

(8) $$R=(R_{ij}{)}_{m\times n}=(W_{j}X_{ij}{)}_{m\times n}$$

(8) Determine the optimal solution Z_ij_^+^ and the worst solution Z_ij_^−^ to form the optimal vector Z^+^ and the worst vector Z^−^, respectively.

(9) $$Z_{ij}^{+}=(maxR_{i1}^{+},maxR_{i2}^{+},maxR_{i3}^{+},\ldots ,maxR_{i10}^{+})$$

(10) $$Z_{ij}^{-}=(maxR_{i1}^{-},maxR_{i2}^{-},maxR_{i3}^{-},\ldots ,maxR_{i10}^{-})$$

(9) Determination of Euclidean spatial distances D^+^ and D^−^ between 14 fertilization treatments and the worst solution.

(11) $$D_{j}^{+}=\sqrt{\sum_{j=1}^{m}{\left[w_{j}\times (r_{ij}-Z_{ij}^{+})\right]}^{2}}$$

(12) $$D_{j}^{-}=\sqrt{\sum_{j=1}^{m}{\left[w_{j}\times (r_{ij}-Z_{ij}^{-})\right]}^{2}}$$

(10) Calculate the comprehensive evaluation value C_i_ of each treatment, i.e., calculate the closeness of the evaluation object to the optimal program as follows:

(13) $$C_{i}=\frac{D_{i}^{-}}{D_{i}^{+}+D_{i}^{-}}$$

### 4.6. Statistics and Analysis of Data

Data were organized using Microsoft Office 2010 and analyzed using IBM SPSS Statistics 21.0. One-way Analysis of Variance (ANOVA) and Least Significant Difference (LSD) tests or Tukey’s Honestly Significant Difference (HSD) tests were performed to compare indicators between different treatment groups, with comprehensive analysis and visualization implemented in Python 3. Pearson correlation analysis was used to assess the associations among leaf, soil, and microbial C, N, and P contents and their stoichiometric ratios. Redundancy analysis (RDA, the gradient length < 3) was performed using Canoco5 to screen key soil factors that affect leaf nutrient factors. The PLS–PM method was used to evaluate factors influencing the yield of *Sapindus saponaria* by the plsdepot (version 0.2.0) and seminr (version 2.3.4) packages in R (version 4.3). The model’s goodness of fit was assessed using the following established thresholds: normed fit index (NFI) > 0.90 and standardized root mean square residual (SRMR) < 0.08, in accordance with Hu and Bentler’s [76] dual-criteria approach. Additionally, the stability of path coefficients was evaluated through a bootstrap analysis with 1000 resamples, employing 95% bias-corrected confidence intervals to test statistical significance.

## 5. Conclusions

This research delved into how balanced nitrogen–phosphorus–potassium (N–P–K) fertilization impacted the nutrient changes in the leaves, the soil, and the soil microbial biomass, along with the yield of the ‘Yuanhua’ variety of *Sapindus saponaria*. The results demonstrated that different fertilization treatments significantly enhanced leaf and soil nutrient contents and soil microbial biomass C, N, and P levels. Utilizing the entropy-weighted TOPSIS model, the N_2_P_2_K_2_ treatment (600 kg·ha^−1^ N, 500 kg·ha^−1^ P, and 400 kg·ha^−1^ K) was identified as the optimal fertilization strategy for maximizing both nutrient content and yield. The best fertilization strategy for this area is to apply N, P, and K to the soil. Applying fertilizers with appropriate N–P–K ratios can remarkably elevate C, N, and P contents of leaf-soil microbes in *Sapindus saponaria* stands and boost the yields of *Sapindus saponaria*. Soil TP, TN, and SOC contents significantly affected leaf nutrient characteristics. Fertilizer application indirectly affected leaf nutrient content by changing soil properties, which in turn had a significant effect on *Sapindus saponaria* yield, especially total soil and leaf P content, which were the main contributors to the yield of *Sapindus saponaria*, and the microbial entropy of soil microorganisms regulated microbial biomass stoichiometric ratios, which further affected *Sapindus saponaria* yield either directly or indirectly. We propose that in the future, investigators should carry out a further examination of the variations in the functional aspects of the microbial community that occur due to the various fertilization treatments and their potential mechanisms of influence on *Sapindus saponaria* yield. Considering that fertilizer application affects soil properties to significantly influence microbial characteristics, which in turn exacerbates or alleviates P limitation and affects plant growth and development, it is reasonable to scientifically formulate fertilizer application strategies to improve soil quality and reduce environmental pollution.

## Figures and Tables

**Table 1:** Comprehensive evaluation results of *Sapindus saponaria* under different fertilization treatments.

	0–20 cm				20–40 cm				
Treatment No.	Euclidean Space Distance D^+^	Euclidean Space Distance D^−^	C_i_	Ranking	Treatment No.	Euclidean Space Distance D^+^	Euclidean Space Distance D^−^	C_i_	Ranking
N_0_P_0_K_0_	0.32	0.03	0.09	14	N_0_P_0_K_0_	0.32	0.04	0.10	14
N_0_P_2_K_2_	0.29	0.07	0.19	13	N_0_P_2_K_2_	0.29	0.05	0.16	13
N_1_P_2_K_2_	0.25	0.12	0.34	11	N_1_P_2_K_2_	0.23	0.11	0.34	12
N_2_P_0_K_2_	0.24	0.11	0.33	12	N_2_P_0_K_2_	0.24	0.13	0.36	11
N_2_P_1_K_2_	0.17	0.20	0.53	5	N_2_P_1_K_2_	0.14	0.22	0.61	2
N_2_P_2_K_2_	0.09	0.27	0.75	1	N_2_P_2_K_2_	0.05	0.30	0.84	1
N_2_P_3_K_2_	0.15	0.21	0.58	3	N_2_P_3_K_2_	0.17	0.23	0.57	3
N_2_P_2_K_1_	0.21	0.15	0.42	10	N_2_P_2_K_1_	0.23	0.15	0.40	9
N_2_P_2_K_0_	0.18	0.23	0.56	4	N_2_P_2_K_0_	0.22	0.14	0.38	10
N_2_P_2_K_3_	0.21	0.16	0.42	9	N_2_P_2_K_3_	0.16	0.20	0.56	6
N_3_P_2_K_2_	0.18	0.19	0.50	7	N_3_P_2_K_2_	0.17	0.21	0.56	5
N_1_P_1_K_2_	0.19	0.20	0.51	6	N_1_P_1_K_2_	0.16	0.21	0.57	4
N_1_P_2_K_1_	0.18	0.25	0.58	2	N_1_P_2_K_1_	0.21	0.22	0.50	7
N_2_P_1_K_1_	0.19	0.16	0.45	8	N_2_P_1_K_1_	0.22	0.15	0.42	8

Note: D^+^ and D^−^ represent the distance between the evaluation object and the positive and negative ideal solution, respectively; Ci is the comprehensive evaluation value.

**Table 2:** Combination of factors and levels in each treatment.

Processing Number	Fertilization Levels (kg·ha^−1^)		
	N	P	K
N_0_P_0_K_0_	0	0	0
N_0_P_2_K_2_	0	500	400
N_1_P_2_K_2_	300	500	400
N_2_P_0_K_2_	600	0	400
N_2_P_1_K_2_	600	250	400
N_2_P_2_K_2_	600	500	400
N_2_P_3_K_2_	600	750	400
N_2_P_2_K_0_	600	500	0
N_2_P_2_K_1_	600	500	200
N_2_P_2_K_3_	600	500	600
N_3_P_2_K_2_	900	500	400
N_1_P_1_K_2_	300	250	400
N_1_P_2_K_1_	300	500	200
N_2_P_1_K_1_	600	250	200

## Data Availability

The original contributions presented in this study are included in the article/Supplementary Materials. Further inquiries can be directed to the corresponding author(s).

## Supplementary Materials

The following supporting information can be downloaded at: https://www.mdpi.com/article/10.3390/plants14091360/s1, Figure S1: Effect of different fertilization treatments on fruit yield of *Sapindus indicus*; Figure S2: Location of the experimental site and experimental design of different NPK treatments; Table S1: RDA of leaf nutrient content influenced by soil properties in different soil layers under different fertilization treatments; Table S2: The contents of soil QC, QN, and QP under different fertilization treatments and soil layers.
