# Supplementary material for: Fertilization Improves the Yield of Sapindus saponaria by Affecting Leaf–Soil–Microbial C–N–P Content and Stoichiometry
Source: Plants (Basel). 2025 Apr 30;14(9):1360. doi: 10.3390/plants14091360 (PMC12073294; doi:10.3390/plants14091360)
Supplement: Supplementary file 1 [file plants-14-01360-s001.zip › plants-3575189-supplementary.pdf]

Fertilization Improves the Yield of Sapindus saponaria by Affecting Leaf–Soil–Microbial C–N–P Content and Stoichiometry

Juntao Liu[1,2 †], Hongbing Yang[3†], Ling Zhou[1], Shangpeng Zhang[4], Jie Chen[5], Xu Wang[5], Shixiong Wu[1], Yingyun Gong[1], Guoqing Zhang[1], Weihua Zhang[2*], Liming Jia[1*]

[1]Key Laboratory of Silviculture and Conservation of the Ministry of Education, College of Forestry, Beijing Forestry University, Beijing 100083, China

[2]Guangdong Provincial Key Laboratory of Silviculture, Protection and Utilization/Guangdong Academy of Forestry, Guangzhou, 510520, China

[3] Key Laboratory of Forest Ecology and Environment of National Forestry and Grassland Administration, Ecology and Nature Conservation Institute, Chinese Academy of Forestry, Beijing 100091, China

[4]Beijing Normal University , Beijing 100091, China

[5]Research Institute of Tropical Forestry, Chinese Academy of Forestry, Guangzhou, 510520, China

Supplementary materials

* Corresponding author, E-mail: jlm@bjfu.edu.cn

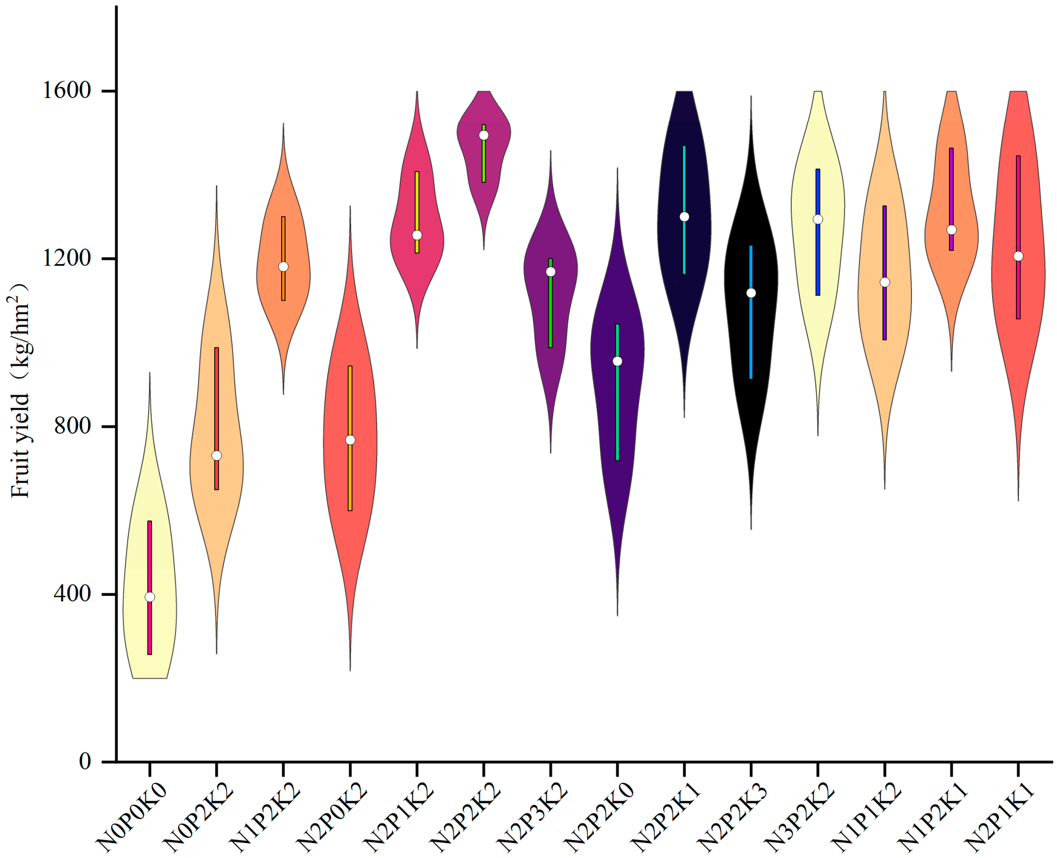

Figure S1 Effect of different fertilization treatments on fruit yield of *Sapindus indicus*

Different lowercase letters indicate significant differences between different treatment groups at 0.05 level.

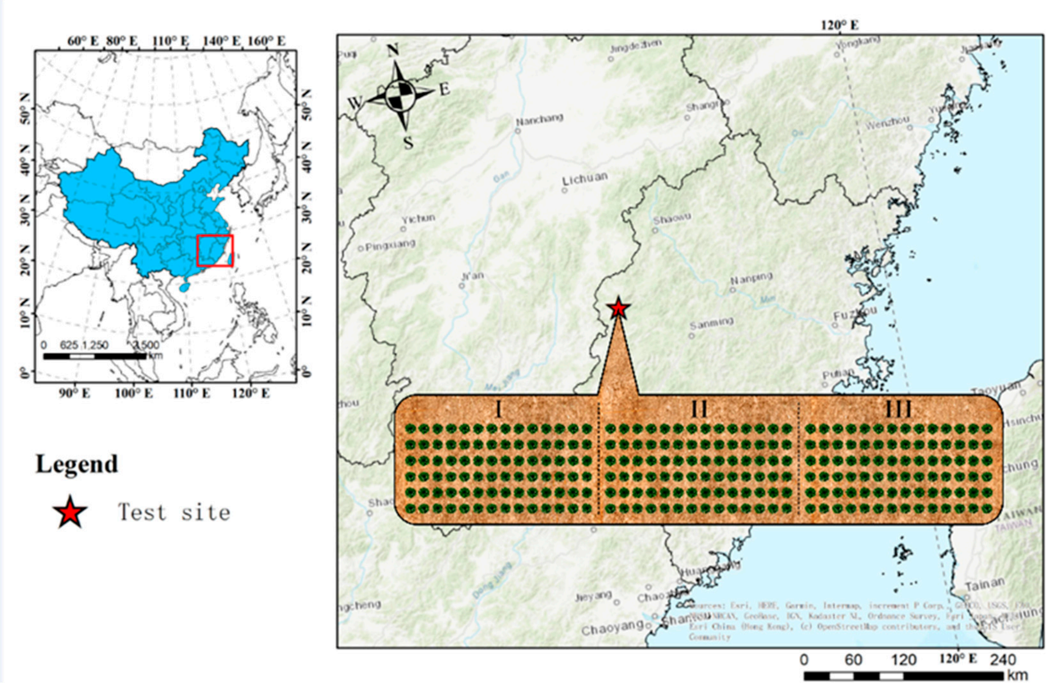

Figure S2 Location of the experimental site and experimental design of different NPK treatments.

* Corresponding author, E-mail: jlm@bjfu.edu.cn

Table S1 RDA of leaf nutrient content influenced by soil properties in different soil layers under different fertilization treatments

Soil properties	0-20 cm		20-40 cm	
	pseudo-F value	*P* value	pseudo-F value	*P* value
SOC	5.9	0.002	7.2	0.006
TN	6.1	0.002	6	0.004
TP	4	0.014	0.9	0.42
N/P	3.1	0.042	0.7	0.52
MBC	2.2	0.122	3.7	0.022
MBP	1.3	0.27	1.1	0.328
MBC/MBP	2.3	0.096	1.9	0.142
MBC/MBN	1.1	0.338	0.3	0.784
MBN/MBP	1.5	0.24	0.3	0.798
MBN	0.8	0.464	1.1	0.354
C/N	0.5	0.636	0.1	0.944
C/P	0.3	0.774	0.8	0.546

The C/N, C/P, and N/P represent the ratios of organic carbon to total nitrogen, organic carbon to total phosphorus, and total nitrogen to total phosphorus, respectively.

* Corresponding author, E-mail: jlm@bjfu.edu.cn

Table S2 The contents of soil QC, QN, and QP under different fertilization treatments and soil layers

Treatments	QC				QN				QP			
	0-20 cm		20-40 cm		0-20 cm		20-40 cm		0-20 cm		20-40 cm	
	Mean	Standard Deviation	Mean	Standard Deviation	Mean	Standard Deviation	Mean	Standard Deviation	Mean	Standard Deviation	Mean	Standard Deviation
$N_0P_0K_0$	0.084	0.009	0.117	0.006	0.126	0.017	0.220	0.010	0.086	0.011	0.093	0.017
$N_0P_2K_2$	0.086	0.008	0.125	0.025	0.110	0.008	0.155	0.019	0.082	0.011	0.075	0.021
$N_1P_2K_2$	0.100	0.008	0.132	0.022	0.106	0.009	0.202	0.061	0.084	0.019	0.090	0.011
$N_2P_0K_2$	0.099	0.013	0.119	0.015	0.133	0.023	0.166	0.014	0.075	0.009	0.083	0.018
$N_2P_1K_2$	0.088	0.008	0.098	0.016	0.134	0.007	0.135	0.012	0.074	0.004	0.077	0.004
$N_2P_2K_2$	0.089	0.002	0.102	0.007	0.131	0.017	0.176	0.051	0.079	0.009	0.102	0.014
$N_2P_3K_2$	0.083	0.009	0.073	0.011	0.104	0.012	0.152	0.023	0.079	0.014	0.112	0.027
$N_2P_2K_1$	0.076	0.008	0.070	0.005	0.117	0.006	0.158	0.011	0.100	0.019	0.111	0.037
$N_2P_2K_0$	0.090	0.005	0.112	0.020	0.123	0.011	0.120	0.019	0.038	0.007	0.066	0.015
$N_2P_2K_3$	0.091	0.007	0.080	0.008	0.146	0.021	0.141	0.018	0.096	0.033	0.088	0.018
$N_3P_2K_2$	0.086	0.008	0.079	0.006	0.131	0.011	0.136	0.007	0.098	0.013	0.081	0.005
$N_1P_1K_2$	0.098	0.007	0.104	0.015	0.098	0.010	0.166	0.046	0.149	0.011	0.112	0.039
$N_1P_2K_1$	0.086	0.015	0.080	0.009	0.116	0.009	0.101	0.014	0.072	0.014	0.075	0.009
$N_2P_1K_1$	0.088	0.004	0.099	0.007	0.116	0.003	0.121	0.010	0.075	0.007	0.070	0.013

The abbreviations QC, QN, QP represent the microbial quotients of carbon (MBC/SOC), nitrogen (MBN/TN), and phosphorus (MBP/TP), respectively.

* Corresponding author, E-mail: jlm@bjfu.edu.cn
